# Comparative effects of MKARE® eggshell membrane and hydrolyzed collagen as nutricosmetics on skin biophysical properties: a randomized clinical trial

**DOI:** 10.3389/fnut.2025.1689701

**Published:** 2026-01-14

**Authors:** Yaiza González-Rodríguez, Manuel A. La Nuez-García, Marina Jiménez, Vega Villar-Suárez, Alejandro Casado-Santos

**Affiliations:** 1 Department of Anatomy, Faculty of Veterinary Sciences, Campus de Vegazana, University of Léon-Universidad de León, Léon, Spain; 2 Arandovo SL, Aranguren, Spain; 3 Labex (Laboratoires Laboratoire d'Expertise Clinique Espagne, S.A.U.), Barcelona, Spain; 4 Institute of Biomedicine (IBIOMED), Faculty of Veterinary Sciences, Campus de Vegazana, University of León-Universidad de León, Léon, Spain; 5 IBIOLEÓN, Hospital of León, Léon, Spain

**Keywords:** Corneometer®, Cutometer®, eggshell membrane, hydrolyzed collagen, skin elasticity, skin flexibility, Tewameter®, TEWL

## Abstract

**Introduction:**

This study aimed to investigate the impact of fresh eggshell membrane (MKARE®) as a nutricosmetic ingredient, with a focus on its influence on skin properties. Biomechanical measurement devices were used to assess the effects on skin condition, and the results were compared with hydrolyzed collagen and a placebo.

**Methods:**

Biophysical parameters were analyzed (using Cutometer®, Tewameter®, Corneometer®, and VISIA®) among participants showing signs of aging. These individuals consumed either 300 mg of fresh eggshell membrane (MKARE®) or 8,000 mg of hydrolyzed collagen, and the results were compared to a placebo group after 28 and 57 days.

**Results:**

The results showed significant improvements in skin firmness/flexibility and elasticity after 57 days of MKARE® intake, compared to both the hydrolyzed collagen and placebo groups.

**Conclusion:**

This study demonstrates that a 300 mg intake of fresh membrane MKARE® positively affects mechanical skin parameters compared to the group that consumed 8,000 mg of hydrolyzed collagen or the placebo group. These improvements suggest that MKARE® consumption regenerates the skin structure, primarily due to positive changes in collagen and elastin formation, which are key contributors to the improvement of skin firmness, elasticity, and barrier protection.

## 1 Introduction

The skin, serving as the first protective barrier of the body, possesses a complex mechanical structure. In recent decades, bioengineering advancements have enabled a precise and objective *in vivo* measurement of the biomechanical properties that define this structure, profoundly revolutionizing our understanding of this organ and its responses to various stimuli (1).

From a biomechanical perspective, the skin can be described as a coexistence and synergy of collagen and elastin networks immersed in a viscous, water-saturated medium rich in glycoproteins and glycosaminoglycans (GAGs) (1–4).

Elastin, comprising 2–4% of the adult dermal dry weight (5, 6), although present in smaller quantities compared to collagen, is crucial for maintaining skin elasticity and flexibility (4). Elastin fibers allow the skin to stretch and return to its original shape after movement, contributing to skin suppleness and preventing wrinkles or sagging. Tropoelastin, the main component of elastin fibers, plays a vital role in skin resilience, wound healing, and dermal regeneration (5). Collagen is the primary structural protein of the skin, comprising approximately 70–80% of its dry weight. It forms a dense, fibrous network within the dermis that provides the skin with its mechanical strength, firmness, and structural integrity. Collagen is often referred to as the “glue” of the skin because it provides a scaffold that maintains the tissue structure and delivers hydration and nutrients to skin cells (8). Hyaluronic acid, one of the most important GAGs in the skin, serves a fundamental function in water retention. Its decline is directly associated with cutaneous dehydration, underscoring its vital function in maintaining skin hydration. In essence, this system can be conceptualized as the mechanical cooperation of the components of the tissue’s extracellular matrix (7, 8).

In recent years, the increasing demand for products that promote skin health and beauty has driven the development of nutricosmetics: nutritional supplements that promise to improve the appearance and function of the skin. However, it is crucial to develop standard and objective methodologies to evaluate their effectiveness. Unlike subjective self-reported questionnaires commonly used in this field, such as the Dermatology Life Quality Index (DLQI) or Skindex-29, biophysical measurements are a powerful tool to quantify changes in skin properties induced by these products.

The fresh eggshell membrane has a rich and complex composition (9, 10), including collagen, hyaluronic acid, and elastin—crucial elements for skin structure. This composition positions MKARE® as a compelling nutricosmetic candidate. While the benefits of hyaluronic acid and collagen for skin health are relatively well described in literature, the specific effects of elastin in this context warrant further investigation.

With aging, the levels of elastin, collagen, and hyaluronic acid progressively decline (11), making supplementation of these components from external sources (12, 13) a valuable strategy for maintaining skin health. Such supplementation has been associated with multiple benefits, including the prevention of sagging and stretch marks, improved skin elasticity and hydration, stimulation of collagen synthesis, strengthening of connective tissues, cartilage, bones, and muscles, reduction in visible signs of aging, and promotion of cellular regeneration (13). Hydrolyzed collagen has also been proven to be an effective alternative as a nutricosmetic. These findings suggest that supplementation with low-molecular-weight collagen peptides can safely reduce skin wrinkling and enhance hydration and elasticity (13).

From a mechanical perspective, the skin functions as a layered composite, exhibiting structural instability under stress, with the most apparent manifestation being the formation of wrinkles (14–17). Biophysical measurements offer an objective and rigorous approach to evaluating the efficacy of nutricosmetics, allowing for the identification of changes induced by these products in the skin and the quantification of their impact on key parameters such as firmness, elasticity, hydration, color, and texture.

For this reason, this study aimed to evaluate the efficacy of MKARE® as a nutricosmetic, specifically assessing its dermo-protective, firming, skin-rejuvenating, and hydrating effects after 4 and 8 weeks of daily use in adults. A comparison was made between MKARE® (300 mg/day), a popular market competitor, hydrolyzed collagen (8,000 mg/day), and a placebo group to demonstrate that this lower dose of MKARE® can achieve comparable or superior objective results.

## 2 Materials and methods

### 2.1 Materials

- MKARE®: MKARE® is available in 300 mg capsules containing fresh eggshell membrane, which is obtained without the use of chemicals and separated through mechanical methods. It is naturally composed of a set of molecules such as collagen, elastin, hyaluronic acid, and more than 400 types of high-value proteins. This product is supplied by Arandovo S.L. and encapsulated by IF3LAB S.L.
- Hydrolyzed collagen: The study utilized hydrolyzed collagen at a dosage of 8,000 mg from a randomly selected commercially available bovine hydrolyzed collagen powder that was unflavored and free of any additional ingredients.
- Placebo: The placebo consists of capsules that contain 300 mg of microcrystalline cellulose and is supplied and encapsulated by IF3LAB S.L.

### 2.2 Pilot study

An initial pilot study was conducted to establish the foundation for designing a larger-scale clinical trial to evaluate the efficacy of MKARE® as a nutricosmetic. This preliminary study served to assess the potential effects of the product, optimize the study design, and enable a better selection of relevant outcome measures.

#### 2.2.1 Inclusion criteria

A total of 15 healthy participants were enrolled at Farmacia Meritxell with dry skin, lack of radiance, and uneven skin tone. Exclusion criteria included reported allergies to eggs or egg products, pregnancy, or breastfeeding. Before data acquisition, all subjects provided written informed consent. Before enrollment, participants were thoroughly instructed on the correct method and timing for product consumption and the proper use of the questionnaires. Treatment group assignments were made using computer-generated randomization tables, resulting in 10 participants allocated to the MKARE® and 5 to the placebo group.

#### 2.2.2 Procedure

Subjects were provided with written instructions and product capsules at the pharmacy. Beginning on day 1 and continuing for 28 days, subjects ingested daily a 300 mg capsule containing either MKARE® or a placebo. The pre-defined primary efficacy endpoint of the study involved measuring biophysical parameters, such as firmness and hydration, using a Cutometer® (Microcaya®) equipped with its designated probes and a Corneometer® 300 (Courage + Khazaka electronic®). Furthermore, a questionnaire was administered to assess improvements in various beauty-related parameters, including skin firmness, hydration, and brightness, as well as hair and nail attributes.

### 2.3 Comprehensive clinical trial

Based on test results from MKARE® on simple biophysical parameters and the subjective evaluation of perceived improvement in the pilot study, a comprehensive study was designed to assess the extent of the effects of MKARE®. MKARE® is a food supplement, not a medicinal product nor a medical device, and no therapeutic effects were attributed to it. Its use remained within the typical consumption doses for healthy subjects, without any medical intervention. Our study falls under food law, such as Regulation (EC) No. 1924/2006 on nutrition and health claims, which requires that claims be supported by generally accepted scientific evidence but does not mandate registration of supporting clinical studies in public databases. The randomized, double-blind, placebo-controlled clinical trial was designed and conducted at the specialized cosmetic laboratory Labex (Laboratoire d'Expertise Clinique Espagne, S.A.U., Barcelona, Spain). This study was conducted in accordance with the Standard Operating Procedures of Laboratoire d'Expertise Clinique Espagne, the protocol signed by the Sponsor, and “in the spirit” of the general principles of Good Clinical Practice (GCP) as published by the ICH (Topic E6: CPMP/ICH/135/95), and the protocol was approved and prospectively registered by the independent Labex Ethics Committee established for study (No. E240181).

#### 2.3.1 Inclusion criteria

Sixty-two healthy female volunteers, aged 40 to 70 years, were recruited. Participants exhibited visible wrinkles, dry skin, lack of radiance, and uneven skin tone, with any facial skin type being acceptable. All subjects received written instructions, and the testing protocol was approved by the regulatory and safety review committee for human testing.

Exclusion criteria included allergy to egg protein or bovine collagen, a history of gastrointestinal diseases or digestive disorders, and the diagnosis of immunological diseases, severe systemic disease, or severe diabetes at study entry. Participants who were pregnant or lactating, or who had a history of alcohol and drug abuse within 6 months before study entry, were also discarded. Furthermore, participants were excluded if they had used any medication, supplementation, or any facial treatment/products to improve facial skin condition before the study, with specific washout periods defined in the protocol. Furthermore, the proportion of volunteers with body skin sensitivity was limited to 60% of the panel (defined as a recent and repeated history of functional symptoms of skin discomfort such as tingling, tightness, warmth, stinging, burning, or redness). The inclusion of subjects with an atopic predisposition was restricted to a maximum of 25% of the panel, consistent with the proportion in the Spanish population.

#### 2.3.2 Study parameters

Randomization: participants were randomized into three groups: fresh eggshell membrane (MKARE®), hydrolyzed collagen, and the placebo group (21, 21, and 20 subjects per product, respectively).

Treatment period: the total treatment period was set to 8 weeks, based on the nutricosmetic composition and previous studies.

Baseline/Follow-up: Measurements were taken before starting the treatment to establish baseline values and repeated on days 28 and 57, according to the study methodology. This choice of sampling times is based on the biological cycle of skin renewal (approximately every 28 days); thus, measurements were taken at this time point to observe improvements in at least two skin renewal cycles (5, 7).

Dosage: The daily dosage was one 300 mg capsule for both the MKARE® and placebo groups. For the hydrolyzed collagen group, an 8-g-per-day dosage was administered using a measuring scoop. Morning was set as the preferred time for administration.

While 300 mg per day of egg membrane has been reported as effective (18), studies have established different effective dosages for collagen: 10 g for non-hydrolyzed collagen and 5 g for hydrolyzed collagen. To ensure robust collagen effectiveness, we opted for an intermediate dosage of 8 g (13, 19).

#### 2.3.3 Study methodology

To comprehensively evaluate the nutricosmetic effects of MKARE® eggshell membrane compared to collagen, a combination of instrumental and subjective assessment techniques was employed to quantify improvements in key skin parameters.

Before data collection, a computer-generated randomization sequence (Excel®) was used at baseline to assign each participant either the left or the right cheek. The measurement area was the center of a 35 cm^2^ (7 cm × 5 cm) surface located on one of the two cheeks of the face. Once the cheek was selected, the same selection was maintained for subsequent measurements (at 28 and 57 days) to ensure correlated and more robust measurements.

Skin biomechanical measurement: Skin viscoelastic properties are evaluated using Cutometer® MPA 580 (Courage + Khazaka electronic®). This device is designed to measure the deformation of the skin area subjected to mechanical suction and its recovery power. Thus, the skin surface is sucked into the probe, and the resulting vertical deformation is measured optically. Changes in light intensity are proportional to the penetration depth and are displayed as curves in a coordinate system (extension/time or pressure/extension). Among the different R parameters that could be measured by this equipment, R0 (skin firmness/flexibility; Uf), R2 (skin elasticity; Ua/Uf), and R6 (skin viscoelasticity; Uv/Ue) were analyzed (Uf: final deformation; Ua: final retraction; Uv: delayed distention; Ue: immediate deformation).

Viscoelastic parameters were analyzed at the center of one cheek of the face.

Skin hydration measurement: Skin hydration is measured using a Corneometer® 300 (Courage + Khazaka electronic®). The average of three corneometric measurements taken at three different points within the defined area is considered the experimental value of skin hydration.

Hydration was measured by assessing electrical capacitance on a randomly selected cheek (left or right) and is expressed in arbitrary units (a.u.). These units are instrument-specific and do not correspond to absolute physical quantities.

- Trans-epidermal water loss measurement: Measurements are performed using a Tewameter® 300 (Courage + Khazaka electronic®). This device is designed to measure the evaporation rate of water diffusing through the outer skin layer, or *stratum corneum* (g/m^2^/h), as well as the relative humidity of the ambient air (%) and the temperature (°C).

Trans epidermal water loss (TEWL: g/m^2^/h) was measured on a randomly selected cheek (left or right).

- Facial wrinkle count: facial wrinkles are counted with a VISIA® module. Detection and counting of facial wrinkles were performed under standard lighting. VISIA® uses high-definition imaging and advanced algorithms to comprehensively assess skin conditions, including wrinkles, providing a thorough evaluation.

All measurements were performed in an air-conditioned room at a constant temperature of 21 °C (+/− 2 °C) and controlled relative humidity (45% ± 5%), after a 30-min rest period to stabilize skin condition.

- Subjective evaluation: The subjective evaluation of the participants was assessed using a 5-point Likert scale questionnaire as a standardized and validated tool. This questionnaire consisted of 10 primary items that measured satisfaction and perceived efficacy of the nutricosmetic across three areas: skin (hydration, radiance, smoothness, firmness, wrinkles, tone, and sagging); hair (thickness, hair loss, shine, and hydration); nails (hardness and growth rate). An assessment of general appearance (skin, hair, and nails) was also included. These questionnaires were developed and validated by the LABEX laboratory, ensuring the reliability of the user perception metrics. This questionnaire was designed for self-administration by subjects at their homes on day 28 of the study. A subsequent 25-question questionnaire was completed before participants’ final visit to Labex to capture a global product appreciation on day 57.

#### 2.3.4 Data processing and statistical analysis

Measurements were performed in triplicate and processed to determine the mean values for each individual at each time point throughout the study. Means and standard errors of the mean (SEM) values were calculated for each group. Normality of distributions was checked using the Shapiro–Wilk test (*p* < 0.05).

For paired values normally distributed, a paired Student’s *t*-test (two-tailed, *p* < 0.05) was performed. Otherwise, the Wilcoxon test (two-tailed, *p* < 0.05) was used. For normally distributed unpaired values, an unpaired Student’s *t*-test (two-tailed, *p* < 0.05) was performed. Otherwise, the Mann–Whitney test (two-tailed, *p* < 0.05) was used. For multiple comparisons, repeated-measures ANOVA or Friedman tests (two-tailed, *p* < 0.05) were used. When applicable, post-hoc comparisons, including appropriate corrections for multiple testing, were performed.

These analyses allowed for the comparison of values across time points (intragroup evaluation) and the determination of statistically significant differences between conditions (intergroup evaluation). GraphPad Software® was used for statistical analysis.

## 3 Results

The full dataset is provided in Supplementary material (descriptive statistics). Data cited in the main text are highlighted in bold and green, data shown in figures are highlighted in bold and blue, and data referenced in both contexts are highlighted in bold with both colors.

### 3.1 Pilot study results

The population recruited in this pilot study consisted of women and men aged 30 to 64 years (mean age 41.13 years).

#### 3.1.1 Biomechanical measurements and subjective perception of the skin

After 28 days of MKARE® or placebo intake, a Corneometer® and a Cutometer® were used to measure hydration and firmness, respectively, and to monitor skin improvement. A significant enhancement of both hydration (34.3 ± 5.11 vs. 44.4 ± 3.93; *p* < 0.05) and firmness (55.10 ± 3.59 vs. 64.60 ± 2.89; *p* < 0.01) was found in the MKARE® group after 28 days compared to the baseline measurements.

**Figure 1:**
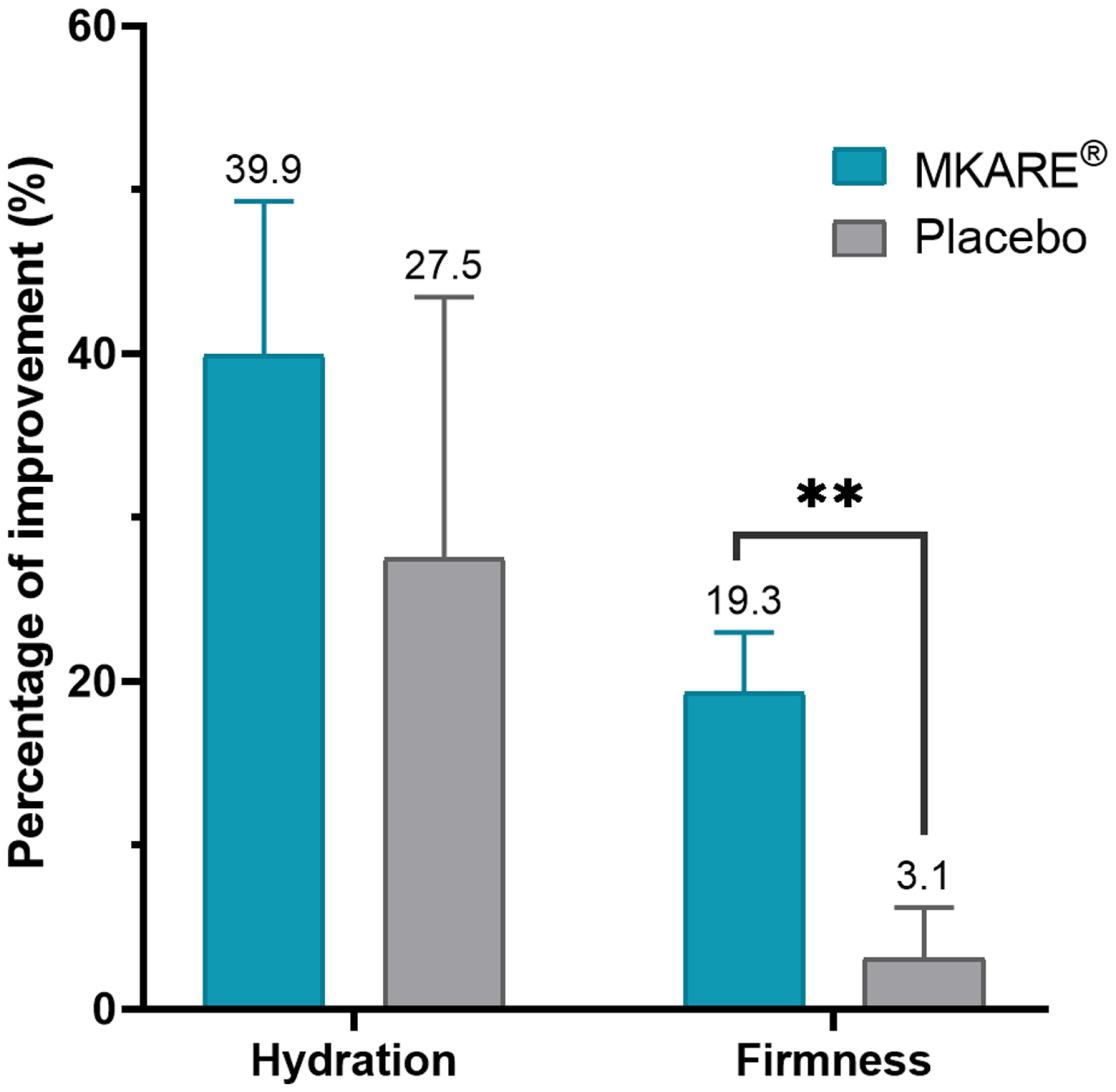
Biophysical improvement after 28 days of treatment for hydration and firmness. Results are shown as the mean percentage of improvement ± SEM. ** *p* < 0.01.

Similarly, the increases in both hydration (+39.9%) and firmness (+19.3%) detected throughout the study showed better performance than the placebo group, although significance was only found in the firmness parameter (*p* < 0.01) (Figure 1).

**Figure 2:**
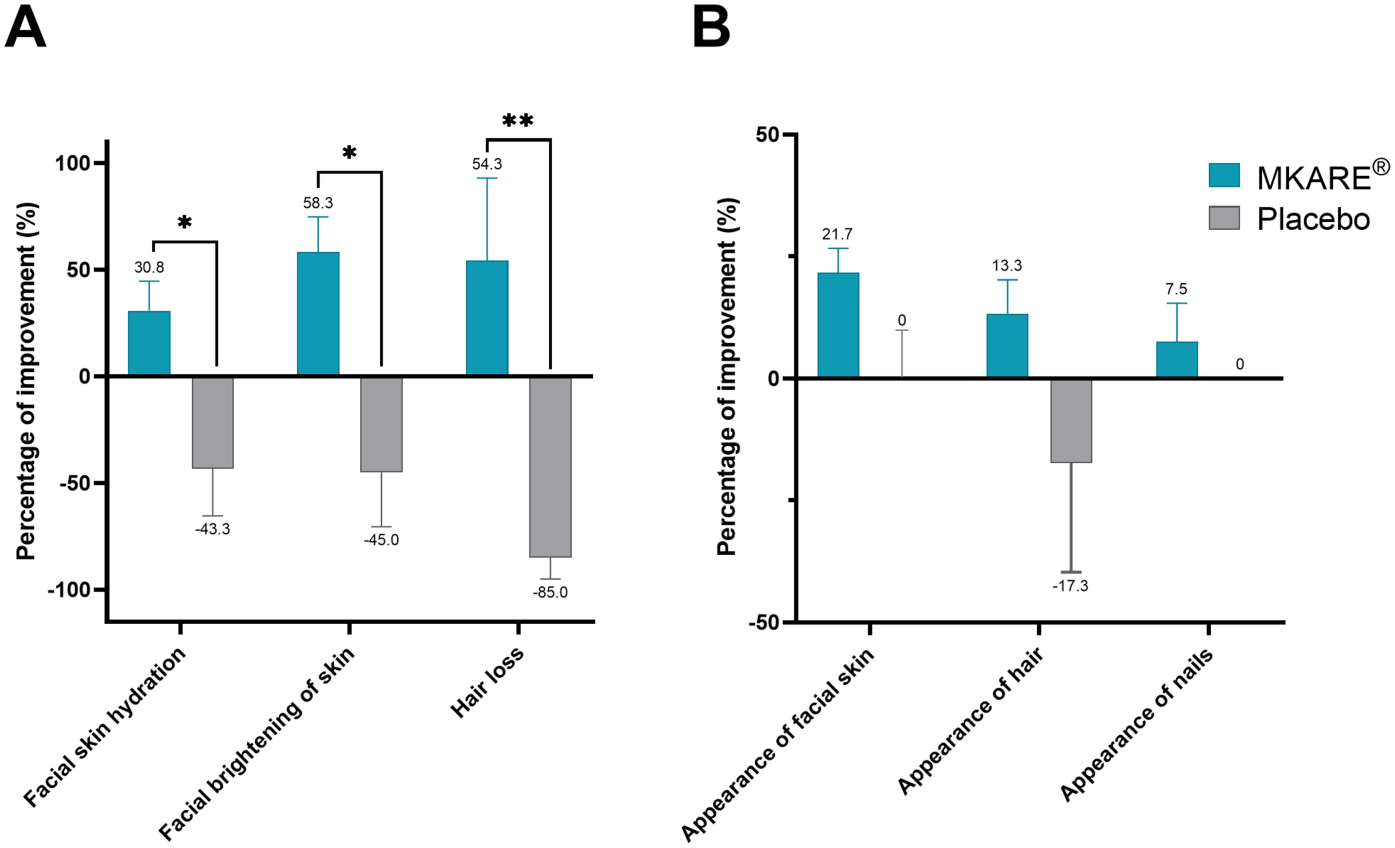
Mean results of subjective perception of improvement (%) at 28 days ± SEM. **(A)** Specific perception of improvement on facial skin hydration, brightening, and hair loss. **(B)** Global perceived improvement in the appearance of skin, hair, and nails. * *p* < 0.05; ** *p* < 0.01.

The pilot study was complemented by a beauty questionnaire to assess the effect of MKARE® on the subjective perception of the participants. Specific perception of improvement (Figure 2A), including facial skin hydration (+30.8%; *p* < 0.05), brightening of the skin (+58.3%; *p* < 0.05), and hair loss (+54.3%; *p* < 0.01), portrayed a significant increase in comparison with the placebo group. Nevertheless, the global perceived results (Figure 2B) showed a remarkable but nonsignificant increase compared to the placebo group.

### 3.2 Comprehensive clinical trial results

**Figure 3:**
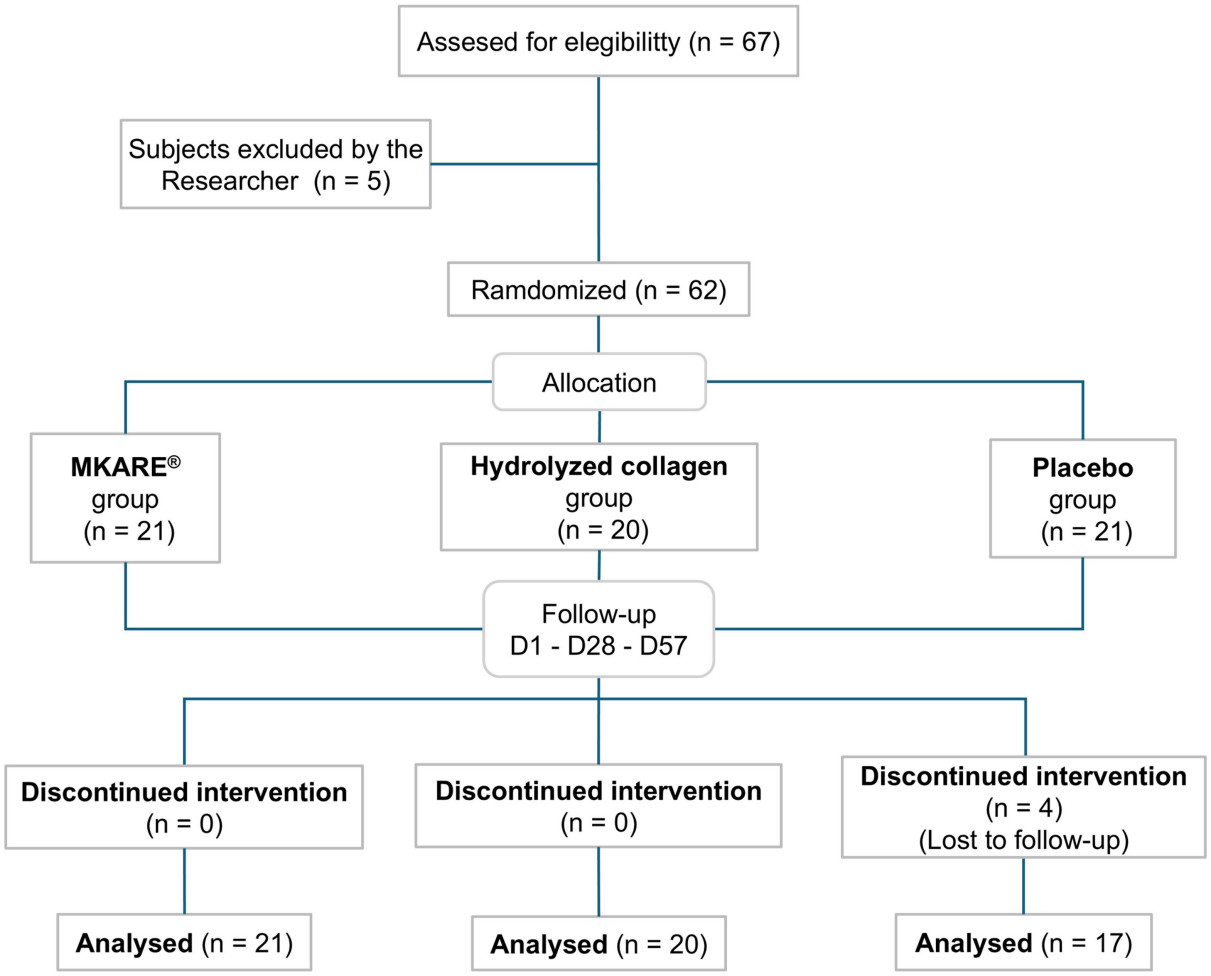
CONSORT flow diagram: Participant enrollment and progress throughout the study.

The study population consisted of women aged from 41 to 69 years old (mean age 53.03 years). Inclusion criteria included self-reported rough and dry skin, reduced luminosity, uneven skin tone, and visible wrinkles. The participant enrollment process is detailed in Figure 3.

#### 3.2.1 Biomechanical measures of the skin: objective perception

Cutometer® measurements taken both before and after the intake of a cosmetic research product allow for an objective assessment of its effect on the biomechanical properties of the skin. The device measures the deformation of a skin area subjected to mechanical suction and its recovery capacity (20, 21). The viscoelastic properties of the skin are directly correlated with its flexibility, elasticity, tone, and firmness.

**Figure 4:**
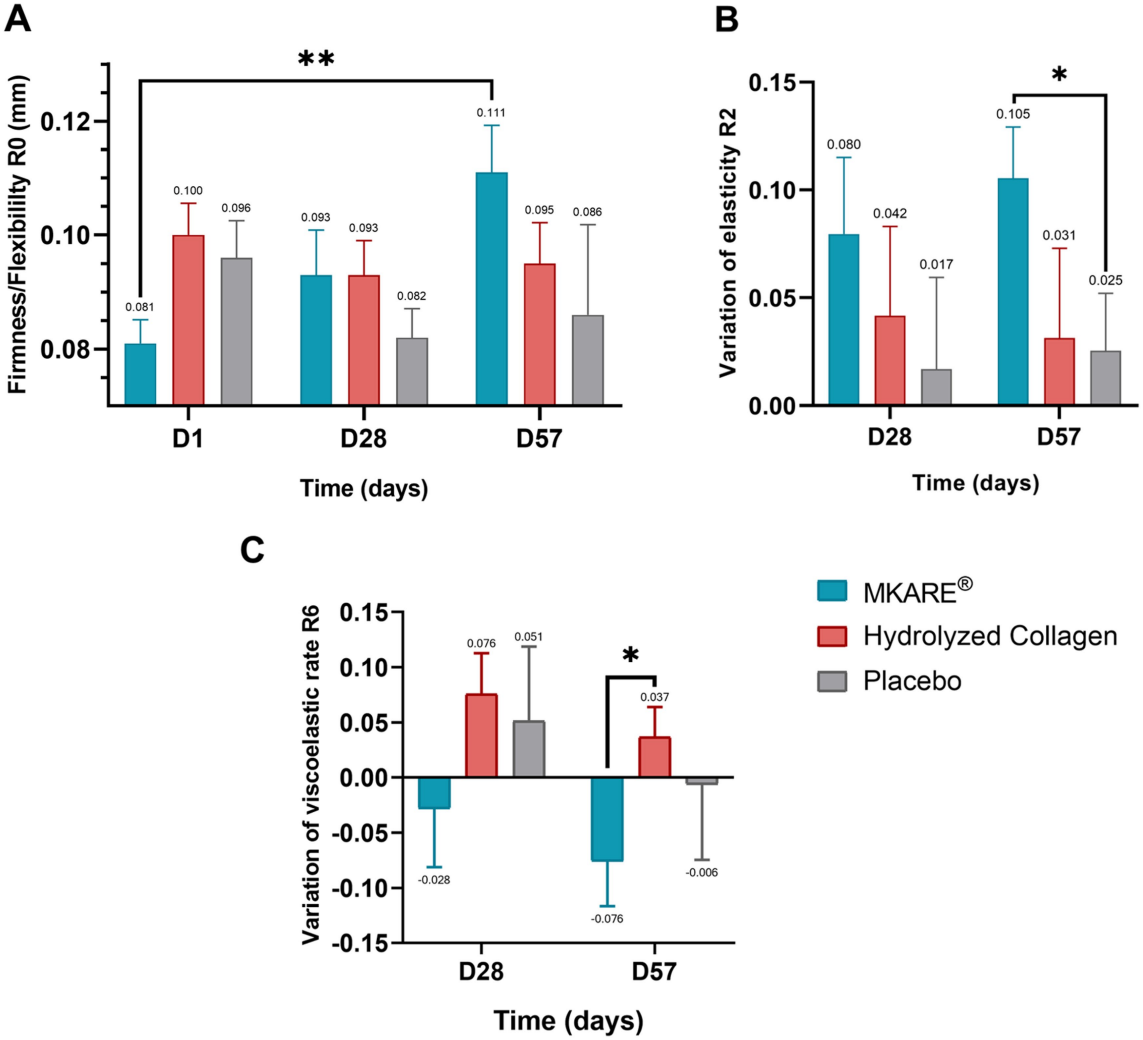
Biomechanical parameters of the tested groups at days 1, 28, and 57 of treatment. **(A)** Mean skin firmness/flexibility (R0) (mm). **(B)** Variation of skin overall elasticity (R2). **(C)** Variation of skin viscoelasticity (R6). Results are shown as the mean value **(A)** or mean of variation value **(B,C)** ± SEM. * *p* < 0.05; ** *p* < 0.01.

For the intergroup analysis, the MKARE® group showed a significant percentage of improvement in skin firmness/flexibility (R0) at 28 days compared to the placebo group (+15.18% vs. −10.81%; *p* < 0.05). Similarly, significant differences were found at 57 days compared to both placebo (+44.73% vs. −3.82%; *p* < 0.01) and hydrolyzed collagen (+44.73% vs. −3.54%; *p* < 0.01). As shown in Figure 4A, a significant increase in R0 values was found for MKARE® in the intragroup analysis at 57 days compared to the baseline (*p* < 0.01). Conversely, the hydrolyzed collagen and placebo groups showed no positive changes in the evaluated parameters for R0.

For overall skin elasticity (R2), a similar pattern was observed, with a significant intragroup increase in MKARE® values at both 28 (0.41 ± 0.03 vs. 0.49 ± 0.03; *p* < 0.05) and 57 (0.41 ± 0.03 vs. 0.52 ± 0.02; *p* < 0.001) days compared to the baseline. Nevertheless, a significant intragroup increase was also observed in the placebo group at 57 days (0.49 ± 0.03 vs. 0.52 ± 0.03, *p* < 0.05). As shown in Figure 4B, the variation (final-initial measurement) of R2 for MKARE® at 57 days was also significantly higher than the placebo group (*p* < 0.05), with an increase of 39.32% from baseline.

Finally, the viscoelasticity rate parameter (R6) portrayed an intragroup reduction for MKARE® in both 28 and 57 days compared to the baseline, with statistical significance in the latter (0.40 ± 0.05 vs. 0.32 ± 0.04; *p* < 0.05) and a significantly lower mean value compared to the placebo group at 57 days (0.32 ± 0.04 vs. 0.38 ± 0.04; *p* < 0.05). Conversely, the placebo group showed no significant change throughout the study, whereas the hydrolyzed collagen group exhibited a significant increase at 28 days (0.31 ± 0.03 vs. 0.39 ± 0.04; *p* < 0.05). Figure 4C shows the development of the viscoelastic rate, showing significant differences between the MKARE® and hydrolyzed collagen groups at 57 days (*p* < 0.05). This trend in R6 provides a complementary perspective to the significant increases in R0 and R2, as well as on the exact effect of the different products on skin properties.

Thus, the increase in R6, a parameter that reflects the balance between viscous and elastic responses and is positively correlated with age, suggests that MKARE® intake improves the elastic profile of the skin.

#### 3.2.2 Effect on skin hydration measurements using Tewameter® and Corneometer®

Transepidermal water loss (TEWL), measured non-invasively with a Tewameter®, assesses the *stratum corneum* barrier function by quantifying water exchange between skin and the environment. Barrier damage increases TEWL, indicating more permeable skin. This method evaluates the protective effect of different products on the skin barrier in adults after repeated applications.

The intragroup evaluation showed a remarkable reduction of TEWL in the MKARE® group at both 28 and 57 days, but significance was only found in the former (12.6 ± 1.41 g/m^2^/h vs. 9.89 ± 0.47 g/m^2^/h; *p* < 0.05) compared to baseline. Similarly, no significant reduction was observed in the hydrolyzed collagen group compared to baseline, although a slight tendency of reduction is appreciable. Conversely, the placebo group showed the worst performance, with a significant increase (*p* < 0.05) in both 28 (12.02 ± 1.47 g/m^2^/h vs. 13.55 ± 1.13 g/m^2^/h) and 57 (12.02 ± 1.47 g/m^2^/h vs. 15.24 ± 1.46 g/m^2^/h) days compared to baseline.

The intergroup comparison showed that the MKARE® values were significantly lower than the placebo group at both 28 (9.89 ± 0.47 g/m^2^/h vs. 13.55 ± 1.13 g/m^2^/h; *p* < 0.01) and 57 (11.39 ± 0.63 g/m^2^/h vs. 15.24 ± 1.45 g/m^2^/h; *p* < 0.05) days but not at the beginning of the clinical trial.

**Figure 5:**
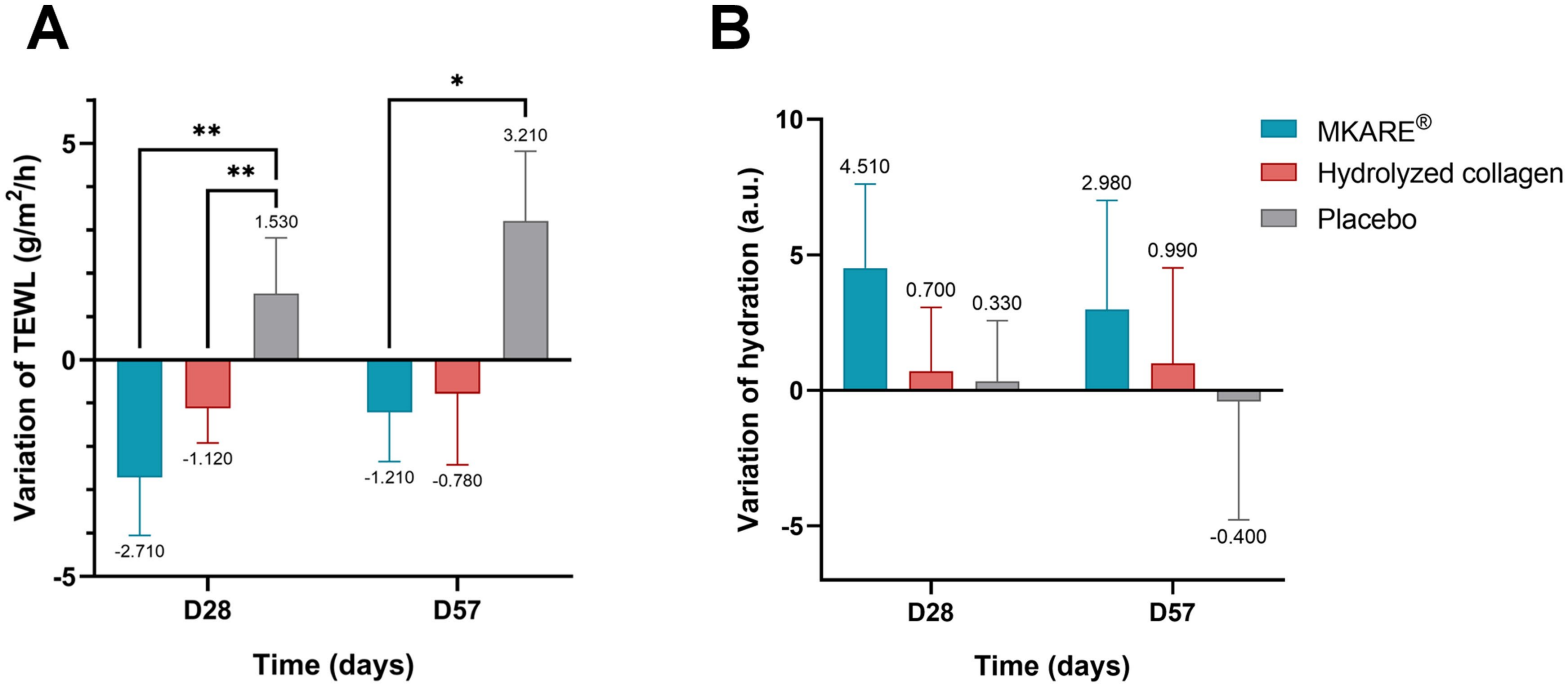
Effect on skin hydration measurements. **(A)** Mean variation of trans-epidermal water loss (g/m^2^/h) ± SEM. **(B)** Mean variation of skin hydration measurements (a.u.) ± SEM. * *p* < 0.05; ** *p* < 0.01.

Figure 5A depicts the variation in TEWL through the trial, with a significant reduction of both MKARE® (*p* < 0.01) and hydrolyzed collagen (*p* < 0.01) compared to the placebo group at 28 days. However, only the MKARE® group showed a significant reduction at 57 days (*p* < 0.05) compared to the placebo.

In contrast to the hydration in the inner skin layer, the *stratum corneum* experienced a modest increase in hydration (10.27 and 8.47% after 28 and 57 days of daily MKARE® consumption, respectively). Although this increase was greater than that observed in the placebo (28 days: 3.77%; 57 days: 0.32%) and hydrolyzed collagen (28 days: 2.67%; 57 days: 2.28%) groups.

Figure 5B shows the variation in hydration throughout the clinical trial with a positive but not significant increase in both the MKARE® and hydrolyzed collagen groups.

#### 3.2.3 Counting of facial wrinkles through VISIA®

**Figure 6:**
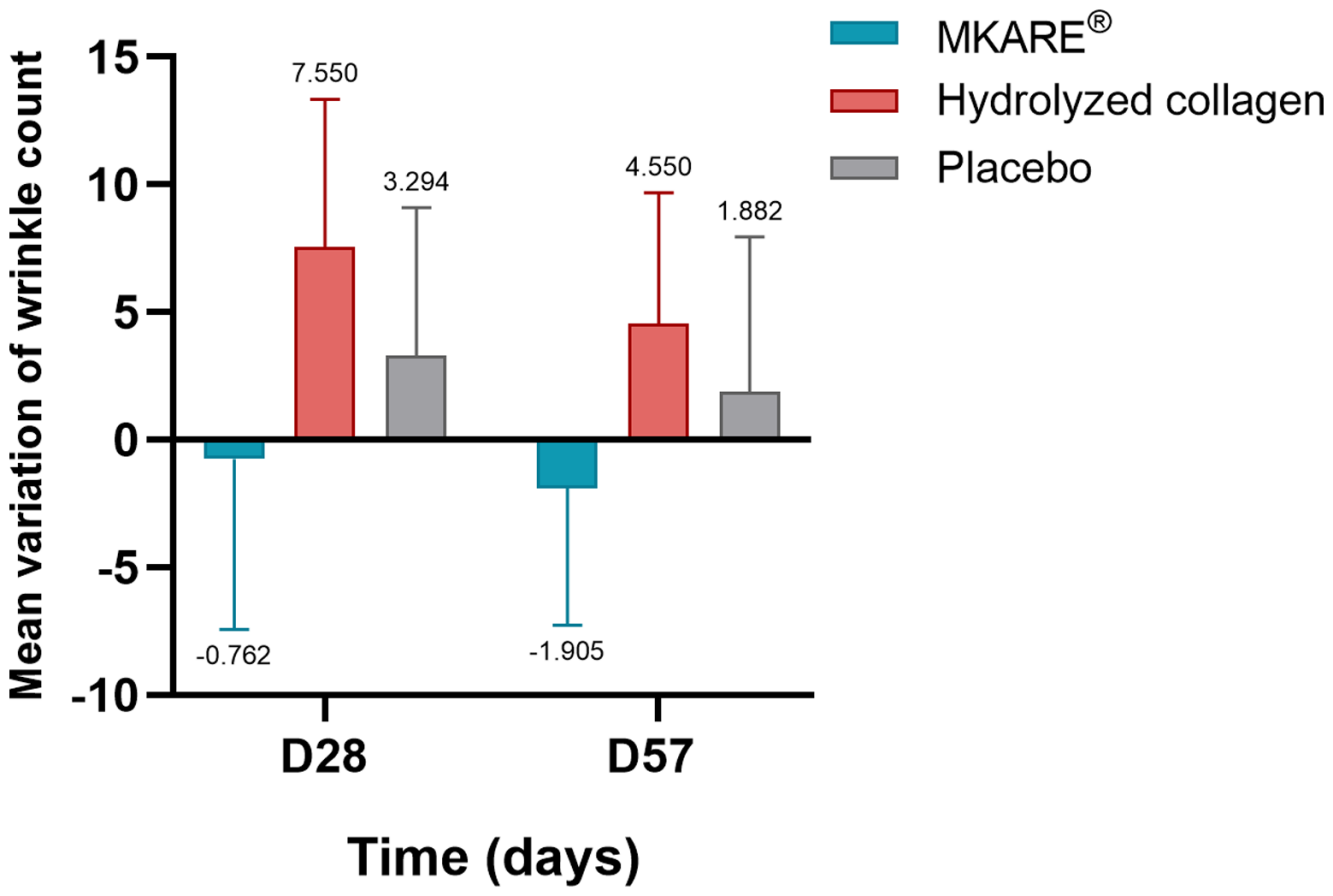
Mean variation of wrinkle count at 28 and 57 days of treatment ± SEM.

Facial wrinkle count was analyzed through VISIA®. Outcomes across the three products show a marginal decrease in wrinkle count in the MKARE® group after 28 (−0.8 ± 6.67) and 57 (−1.9 ± 5.35) days of treatment. On the contrary, both the hydrolyzed collagen (28 days: 7.5 ± 5.80; 57 days: 4.5 ± 5.13) and placebo (28 days: 3.3 ± 5.8; 57 days: 1.9 ± 6.06) groups showed a slight increment of this parameter. Although not significant, these results demonstrate the promising potential of MKARE® intake to reduce the number of wrinkles (Figure 6).

#### 3.2.4 Subjective evaluation

The distribution results were not normal, so the statistical analysis was carried out using the Friedman test. Results from the specific questionnaire show that the MKARE® group consistently achieved superior performance across all measured parameters when compared to both the hydrolyzed collagen and placebo groups.

• Skin hydration: The MKARE group improved by 2.0 points (40%) (*p* < 0.001), outperforming the hydrolyzed collagen group’s 1.65 points (33%) and the (*p* < 0.001) placebo group’s 1.0 points (20%) (*p* < 0.01).

• Facial wrinkle reduction: The MKARE group achieved a 1.71-point improvement (34.4%) (*p* < 0.001), compared to 1.50 points (30%) (*p* < 0.001) for hydrolyzed collagen and 0.76 points (15.4%) (*p* < 0.05) for the placebo group.

• Skin sagging: The MKARE group improved by 1.62 points (32.4%) (*p* < 0.001), while the hydrolyzed collagen group improved by 1.20 points (24%) (*p* < 0.001) and the placebo group by 0.88 points (17.6%) (*p* < 0.05).

• Hair loss: The MKARE group experienced a reduction of 0.38 points (−12.1%) (*p* > 0.05), a significant finding given that the hydrolyzed collagen group showed an increase of 0.15 points (+4%) (*p* > 0.05) and the placebo group increased by 0.35 points (+10.1%) (*p* > 0.05).

Similar results from the overall subjective questionnaires demonstrated the superiority of MKARE® compared to both hydrolyzed collagen and the placebo group.

Specifically, by day 57, the MKARE® group achieved an overall facial skin assessment of 1.67 points (33.2%) (*p* < 0.001), outperforming the hydrolyzed collagen group, which recorded 1.35 points (27%) (*p* < 0.001), and the placebo group, which showed 0.88 points (17.6%) (*p* < 0.05).

Similarly, for hair assessment on day 57, the MKARE®-consuming group attained 1.67 points (33.4%) (*p* < 0.001). The hydrolyzed collagen group recorded 1.40 points (28%) (*p* < 0.001), while the placebo group was 1.24 points (24.6%) (*p* < 0.01).

## 4 Discussion

Recent advances in nutricosmetic research have opened new perspectives for noninvasive strategies to support skin health, particularly through bioactive compounds derived from functional food ingredients. In this context, the present randomized, double-blind, placebo-controlled clinical trial provides evidence that fresh eggshell membrane (MKARE®) exerts measurable benefits on key biophysical parameters of the skin when compared with both hydrolyzed collagen and placebo. By combining objective instrumental measurements (Cutometer®, Tewameter®, Corneometer®, VISIA®) with validated subjective questionnaires, the study demonstrates that MKARE® improves skin firmness, elasticity, barrier function, and perceived appearance across multiple outcome domains. In the pilot phase, MKARE® intake led to a nearly 20% increase in firmness and an approximately 40% rise in hydration, outperforming placebo in firmness and showing a clear trend toward superior hydration despite variability in the control group. These preliminary findings were confirmed and expanded in the comprehensive clinical trial, where MKARE® produced the most robust improvements across biomechanical and barrier-related outcomes compared with hydrolyzed collagen and placebo.

The pilot study results revealed that MKARE® significantly increased firmness by almost 20%, compared to the mere 3% increase observed in the placebo group. Similarly, a ≃ 40% increase in hydration was registered with MKARE®, although it was not significantly superior to placebo (≃28%), mainly due to the high variability observed in the latter. In the comprehensive trial, MKARE® produced a 44.73% increase in R0 at day 57 and a nearly 40% increase in R2, whereas the hydrolyzed collagen and placebo groups did not show comparable changes. Since R0 reflects firmness and extensibility (20–22) and R2 represents the ratio of total retraction to maximum deformation, they provide a comprehensive measure of overall skin elasticity (23). These findings indicate that MKARE® enhances both the skin’s ability to deform under stress and its capacity to recover its original shape, features typically associated with younger, better-organized dermal matrices. The concomitant reduction in R6 further supports a shift toward a more elastic and less viscous biomechanical profile, consistent with a rejuvenated mechanical behavior of the skin.

**Figure 7:**
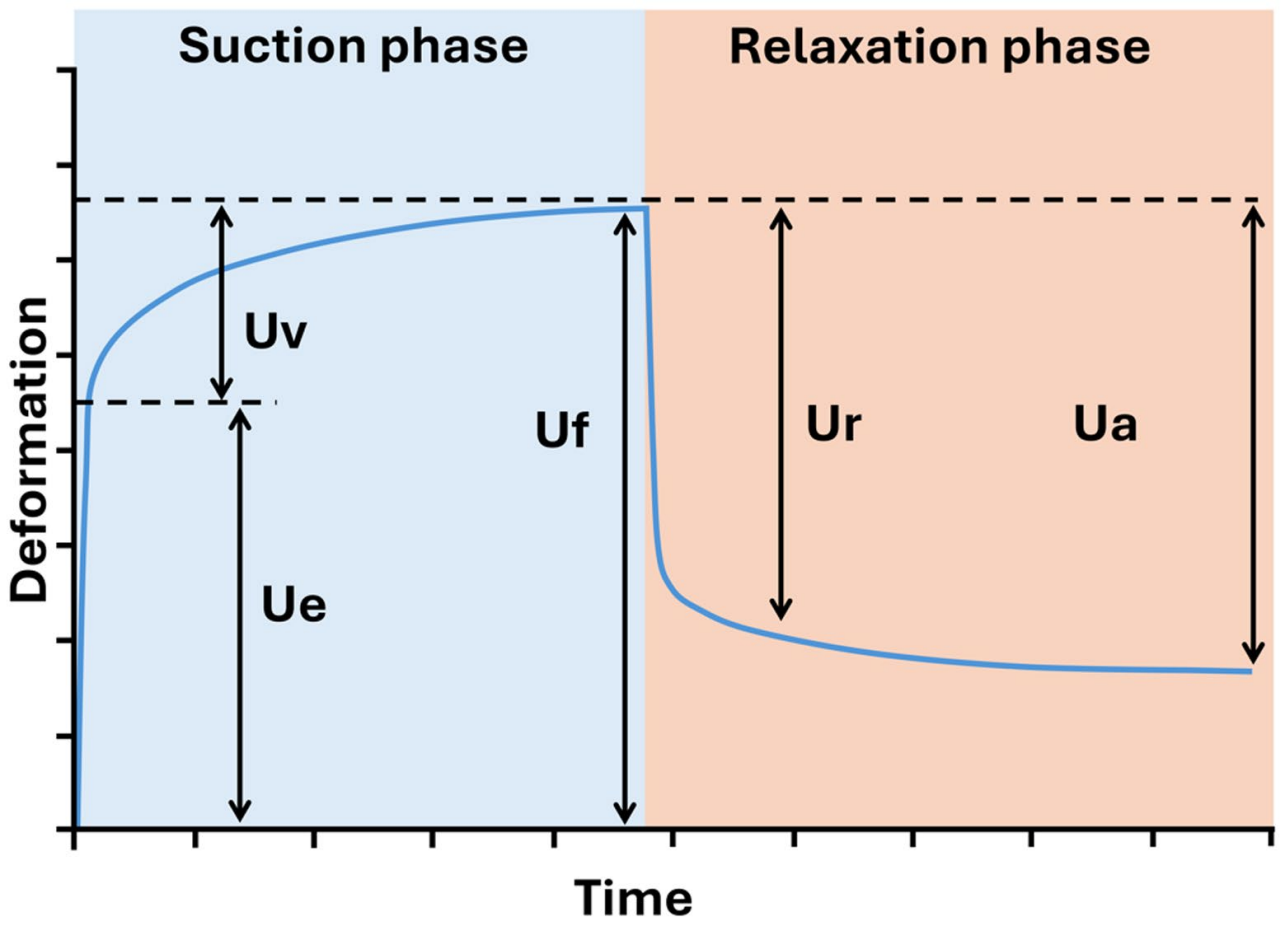
Skin deformation pattern over time using a Cutometer®. Ue, immediate deformation; Uv, delayed distention; Uf, final deformation; Ur, immediate retraction; Ua, final retraction; Ua–Ur, delayed retraction.

The decline in cheek skin elasticity is a significant concern for the middle-aged population, promoting extensive research on cosmetic interventions targeting age-related changes in this area. Cutometer®-derived parameters offer a mechanistic link between MKARE® intake and the observed clinical improvements in skin quality (20, 23, 24), supporting the interpretation that the changes measured in R0, R2, and R6 reflect meaningful restoration of age-related biomechanical losses rather than short-lived cosmetic effects. Previous studies have reported age-related declines in Cutometer® parameters and have linked higher R0 and R2 values to younger, more elastic skin, which reinforces the interpretation that MKARE® contributes to a partial restoration of age-related losses in dermal biomechanics rather than a purely transient cosmetic effect. In studies by Ohshima et al. (23), a decrease in R0 and R3 was observed in facial skin with age. Younger skin profiles typically display higher Ue values, reflecting greater elastic recovery. Given that R0 is the sum of Ue and Uv (Figure 7), younger skin tends to have larger R0 values (25). Furthermore, studies indicate that moisturizers or emollients can elevate both Ue and Uv by promoting skin softening and increasing epidermal plasticity (23–26). The significant increase in R0 we observed with MKARE®, alongside the improved R2, suggests that MKARE® enhances skin firmness, extensibility, softening, and elasticity. This finding corroborates the report from Chen et al. of a significant negative correlation between R0 and age (25), implying that higher R0 values are associated with younger skin. Thus, the increased R0 observed at both 28 and 57 days following MKARE® consumption suggests a potential skin-rejuvenation effect.

While some of these results did not reach statistical significance, the consistent trend toward improvement observed in the MKARE® group suggests a positive effect on skin structure. This conclusion is supported by the biological credibility of the ingredient, since eggshell membrane provides key components of the dermal matrix, such as Type I collagen, hyaluronic acid, and elastin (12, 22). Thus, MKARE® supplementation is postulated to directly influence the regeneration and maintenance of these endogenous biomolecules, providing essential systemic precursors that can enhance skin barrier function and biomechanical properties. By supplying these naturally occurring constituents in a bioavailable form, MKARE® may promote the restoration of dermal architecture and contribute to improvements in skin firmness, hydration, and elasticity observed in our study.

Notably, elastin is a key structural protein that provides elasticity and resilience to tissues and plays a crucial role in maintaining the skin’s ability to recover its flexibility and youthful appearance (4). This process begins with fibroblasts synthesizing pro-elastin from its specific amino acid components. The pro-elastin then undergoes a complex series of extracellular modifications and assemblies, ultimately forming mature elastin fibers that are essential for supporting the structural integrity and elasticity of the skin (2, 5, 6). Given its critical function, the adequate availability of elastin-specific amino acids and related cofactors may support the synthesis and proper assembly of elastin fibers, potentially contributing to the restoration and maintenance of skin elasticity (12).

Collagen is a fundamental structural protein in the skin that provides tensile strength to the dermis, but its functionality depends on a well-organized assembly of fibers within the extracellular matrix. Collagen fibers form a dense, interconnected network with elastin, which together underpin the elasticity and resilience of the skin. Critically, this structural integrity requires hydration, supplied by hyaluronic acid, an essential component that maintains tissue hydration, enables nutrient exchange, and optimizes the functional arrangement of both collagen and elastin fibers (7). In light of our results, the MKARE® group demonstrated superior improvements in biophysical skin parameters compared to the hydrolyzed collagen group, suggesting that the benefits observed are not solely attributable to collagen supplementation. The synergistic profile of MKARE® provides a range of extracellular matrix cofactors, including amino acids from collagen, hyaluronic acid, glycosaminoglycans, and notably, a rich supply of elastin, that together promote optimal dermal homeostasis and regeneration (27, 28). The bioactive fragments resulting from MKARE® digestion may simultaneously stimulate fibroblasts to enhance endogenous synthesis of collagen, elastin, and HA, offering a comprehensive approach to skin restructuring that extends beyond the focused effects of hydrolyzed collagen alone. This may explain why the magnitude of improvement seen in the MKARE® group was not replicated in the placebo or hydrolyzed collagen groups, a finding consistent with previous evidence on the need for multi-component intervention to support skin integrity and elasticity (29).

The most pronounced effect of MKARE® on skin hydration is observed in the deeper layers, indicating a primary action at the structural level. This is likely attributable to the rich composition in collagen, hyaluronic acid, and elastin of the eggshell membrane, key components also found in skin (1, 5–7). Furthermore, *in vitro* studies with chondrocytes have demonstrated that MKARE® induces the production of aggrecans and collagen, further supporting its regenerative potential (18). These *in vitro* findings provide a mechanistic explanation for the positive changes observed in the mechanical parameters of the skin as measured by cutometry.

Skin capacitance, commonly measured with a Corneometer®, evaluates the water content of the upper epidermal layers to quantify product hydration effects. Due to the high dielectric constant of water, the electrical properties of the *stratum corneum* depend on its water content, enabling investigation of upper epidermal hydration levels. Therefore, repeated capacitance measurements objectively assess the effect of a product on skin hydration levels compared to baseline values. TEWL is considered the most important inside-out water skin barrier parameter, indicating the integrity of the *stratum corneum* (30). The results obtained for the MKARE® group on both 28 and 57 days clearly demonstrate a high reduction in the TEWL. This suggests that the evaluated product provides a protective effect on the skin barrier, while the placebo group showed an increase in TEWL. This increase is likely influenced by the timing of the clinical study (May–June), which corresponds to the beginning of summer in Spain, characterized by increased solar intensity and greater potential for sun exposure. Therefore, the results clearly indicate that MKARE® intake protects the skin barrier function compared to individuals who do not consume any protective product.

The results of this study provide compelling evidence supporting the efficacy of MKARE® in promoting key aspects of skin health. While the biophysical measurements objectively demonstrated significant improvements, particularly in hydration, wrinkle attenuation, and skin firmness, the subjective assessments further reinforced these outcomes. Because TEWL primarily reflects *stratum corneum* barrier integrity, this pattern indicates that MKARE® strengthens the epidermal barrier under environmental conditions that typically promote barrier deterioration, while the concurrent gains in Cutometer® parameters point to a parallel reorganization of the dermal matrix. The simultaneous improvement in barrier function (lower TEWL) and biomechanical behavior (higher R0 and R2, lower R6) therefore acts as an integrated surrogate marker of a broader regenerative effect on cutaneous structural matrices, rather than an isolated hydration response. These findings are consistent with the bioactive composition of MKARE eggshell membrane, which contains hyaluronic acid, glycosaminoglycans, and collagen, all of which are systemically absorbed (31).

Moreover, increased flexibility implies a reduced susceptibility of the skin to rupture under stress (20). Enhanced elasticity is indicative of the ability of the skin to return to its original state after deformation, thereby minimizing the formation of wrinkles, such as facial expression lines that can appear with age (11). The combined improvements in R0 and R2 observed in our study indicate that the consumption of MKARE® effectively enhances both the flexibility and elasticity of the skin, parameters that are likely correlated with the nature and composition of elastin fibers in the skin. Notably, the effects observed in the MKARE® group exceeded those in both the hydrolyzed collagen and placebo groups, suggesting that MKARE® may offer added benefits beyond conventional collagen supplementation. The concordance between objective and perceived improvements underscores the clinical relevance of the results, highlighting not only the biological impact of the intervention but also its tangible perception by users. This alignment is particularly important in dermatological and cosmetic interventions, where user satisfaction and perceived efficacy play critical roles in long-term adherence and real-world effectiveness.

It should be noted that the population included in this trial was not stratified by age, which may represent a limitation when interpreting results related to age-dependent changes in skin structure. Nevertheless, all treatment groups were designed to be as homogeneous as possible, and no significant differences in mean age were detected, as the sample was predominantly composed of postmenopausal women, a biologically distinct population in whom collagen and elastin synthesis is already reduced. Given that age is a well-recognized determinant of cutaneous biomechanics and hydration capacity, future studies specifically comparing younger and older cohorts would allow a more precise characterization of MKARE® efficacy across different stages of skin aging.

Although the exclusion criteria were designed to minimize the enrollment of participants using medication or other skin-influencing treatments, several factors remain difficult to fully control in human intervention studies. Potential confounding factors, such as quality of life, chronic stress, and sleep quality, may have influenced baseline condition and responsiveness of the skin, as both psychosocial status and sleep patterns are known to modulate systemic inflammation and cutaneous barrier function. Similarly, individual differences in dietary habits, including micronutrient intake like vitamin C, zinc, and copper, which are essential cofactors for collagen and elastin synthesis, could not be standardized across participants. Environmental exposures, particularly to sunlight and air pollution, might also have varied considerably despite restricting the study period to late spring and early summer. Finally, inherent anatomical and physiological differences in the skin, including variations due to aging (chronoaging vs. photoaging) and baseline heterogeneity in anatomical sites, may have affected the response to supplementation despite age-matching across groups. These limitations should be considered when interpreting the results and highlight the need for future studies that incorporate more exhaustive monitoring and control of lifestyle and environmental variables to clarify further the effects observed.

## 5 Conclusion

Based on the results of this study, MKARE®, composed of fresh eggshell membrane, shows a great alternative as a nutricosmetic. The results reveal that a dose of only 300 mg of MKARE® demonstrated superior efficacy compared with 8,000 mg of hydrolyzed collagen in objective and subjective measures. The mechanical properties of skin primarily depend on the dermal and hypodermal collagen and elastic fiber network that is embedded in a viscous ground matrix, to which the epidermal layer also contributes. Significant improvements in skin firmness/flexibility and elasticity were observed with the intake of MKARE® compared to both placebo and hydrolyzed collagen groups. The reduction in water loss from the deeper dermal layers, observed in the MKARE® group, represents another effect intrinsically connected to the biomechanical structure of the skin. Furthermore, this enhanced deep hydration is strongly correlated with increased skin flexibility, as greater hydration facilitates the movement of internal structures.

Taken together, these findings suggest that MKARE® may serve as a superior alternative for supporting skin health, with both measurable and perceivable benefits. However, future research should aim to confirm these findings over extended periods of time and to better understand the biological pathways through which MKARE® exerts its effects.

## Data Availability

The raw data supporting the conclusions of this article will be made available by the authors, without undue reservation.
