# Supplementary material for: Comparative effects of MKARE® eggshell membrane and hydrolyzed collagen as nutricosmetics on skin biophysical properties: a randomized clinical trial
Source: Front Nutr. 2026 Jan 14;12:1689701. doi: 10.3389/fnut.2025.1689701 (PMC12846929; doi:10.3389/fnut.2025.1689701)
Supplement: Supplementary file 1 [file Table_1.DOCX]

1. Was it easy to incorporate the use of this product into your daily routine?
2. Overall rating of the product.
3. Did you notice any unwanted side effects after using the product?
4. Would you recommend the product?
5. Would you buy the product?
6. Reason(s) why I don't want to buy it.
