# Supplementary material for: Comparative effects of MKARE® eggshell membrane and hydrolyzed collagen as nutricosmetics on skin biophysical properties: a randomized clinical trial
Source: Front Nutr. 2026 Jan 14;12:1689701. doi: 10.3389/fnut.2025.1689701 (PMC12846929; doi:10.3389/fnut.2025.1689701)
Supplement: Supplementary file 2 [file Table_2.DOCX]

The items provided will all be rated on a fixed five-point Likert scale (ratings vary from: 0 very unsatisfied to 5 very satisfied).

**​** **Questionnaire at t = 28, t = 57:**

**I. Rate the improvements experienced in the following aspects of your skin from 0 to 5 (0 = very little improvement, 5 = great improvement):**

1. Facial skin hydration
2. Facial skin brightness
3. Facial skin smoothness
4. Facial skin firmness
5. Facial skin wrinkles
6. Facial skin tone
7. Skin sagging
8. Overall rating of the appearance of your facial skin

**II. Rate the improvements experienced in the following aspects of your hair from 0 to 5 (0 = very little improvement, 5 = great improvement):**

1. Hair thickness
2. Hair loss
3. Hair shine
4. Hair hydration
5. Overall condition of your hair

**III. Rate the improvements experienced in the following aspects of your nails from 0 to 5 (0 = very little improvement, 5 = great improvement):**

1. Nail hardness (0 = very soft, 5 = very hard)
2. Nail growth speed (0 = very slow growth, 5 = very fast growth)
3. Overall appearance of your nails (0 = very poor appearance, 5 = very good appearance)
